# Impact of bedaquiline regimen on the treatment success rates of multidrug-resistant tuberculosis patients in Egypt

**DOI:** 10.1038/s41598-024-65063-8

**Published:** 2024-07-15

**Authors:** Magda Afifi, Wagdy Amin, Dina Helal, Rasha Ashmawy, Yousra A. El-Maradny, Noha Khalifa, Ramy Mohamed Ghazy

**Affiliations:** 1 General Administration of Chest Diseases, MoHP, Cairo, Egypt; 2 Chest Diseases Consultant and MDR Coordinator/NTP, Cairo, Egypt; 3 Department of Biomedical Informatics and Medical Statistics, Medical Research Institute, Alexandria University, Alexandria, Egypt; 4 Department of Clinical Research, Maamora Chest Hospital, MoHP, Alexandria, Egypt; 5 Clinical Research Administration, Directorate of Health Affairs, MoHP, Alexandria, Egypt; 6 Pharmaceutical and Fermentation Industries Development Center, City of Scientific Research and Technological Applications (SRTA-City), New Borg EL-Arab, Alexandria, 21934 Egypt; 7 Family and Community Medicine Department, College of Medicine, King Khalid University, Abha, Saudi Arabia; 8 Tropical Health Department, High Institute of Public Health, Alexandria University, Alexandria, Egypt

**Keywords:** Tuberculosis, Infectious diseases, MDR-TB, Antibiotic resistance, Bedaquiline, Egypt, Microbiology, Antimicrobials, Clinical microbiology

## Abstract

Bedaquiline (BDQ), an innovative anti-tuberculous (TB) agent, has attracted attention for its potential effectiveness against drug-resistant TB. This study investigated the impact of BDQ-containing regimens on treatment success rates among multi-drug resistant tuberculosis (MDR-TB) patients in Egypt. We conducted a prospective cohort study that included all adult non-pregnant patients treated in MDR-TB centers in Egypt from April 1, 2020, to June 30, 2021, with follow-up extended until December 31, 2022. The study compared patients prescribed BDQ according to national protocols with those receiving conventional treatments for MDR-TB. Treatment success rates, mortality rates, and adverse events were analyzed using descriptive statistics, chi-square tests, logistic regression, and Kaplan–Meier survival curves. Adjustment for potential confounders was conducted using propensity score matching and Cox-hazard regressions. A total of 84 patients were included in this study. The median age of the study participants was 39 years; 22.6% were women, 57.1% were unemployed or housewives, and 1.2% had human immunodeficiency virus (HIV). Regarding the treatment regimen, 67.8% were exposed to BDQ-based treatment. Among the 55 patients (65.5%) with treatment success, a significantly higher success rate was observed in the BDQ group (73.7%) compared to the conventional group (48.1%), P = 0.042. Additionally, the incidence of skin discoloration was significantly higher in the BDQ group compared to the conventional group (38.6% versus 0.0%, P < 0.001). Despite the lower mortality incidence in the BDQ-group (14.0% versus 22.2% in the conventional group), the Kaplan–Meier survival analysis revealed no excess mortality associated with the BDQ-group, with a hazard ratio (HR) of 0.62 (95% CI 0.21–1.78, P = 0.372). Propensity score matching, while considering factors such as lesion site, diabetes mellitus, hepatitis C virus, and smoking, revealed a significant increase in the success rate associated with BDQ inclusion, with an HR of 6.79 (95% CI 1.8–25.8). In conclusion, BDQ is an effective and tolerable medication for treating MDR-TB, associated with lower mortality rates compared to conventional treatment.

## Introduction

Tuberculosis (TB) is an infectious disease caused by *Mycobacterium tuberculosis* (MTB) and presents a significant public health challenge. The disease spreads when infected individuals release the bacteria into the air, usually by coughing, and can affect the lungs (pulmonary TB) or other parts of the body (extra-pulmonary TB)^1^. TB is a major contributor to global mortality, ranking among the top ten causes of death worldwide in 2018. Approximately half a million people develop multi-drug resistant tuberculosis (MDR-TB) annually^2^.

Standard treatment for susceptible TB involves a six-month regimen of four antimicrobial agents, which are considered first-line drugs: rifampicin, isoniazid, pyrazinamide, and ethambutol^3^. However, MTB can develop resistance to these drugs, leading to treatment failure. Resistance can be limited to rifampicin (RR-TB) or extend to other first-line drugs and become MDR-TB. Where, RR-TB refers to the inability of MTB to respond to rifampicin treatment, either alone or in combination with other first-line anti-TB medications. MDR-TB is the resistance of MTB to treatment with rifampicin and isoniazid, which are the most powerful first-line anti-TB medications^2,3^. Treating RR/MDR-TB is challenging due to limited treatment options, expensive medications, and longer treatment durations compared to standard drug-sensitive TB treatment. Furthermore, patients typically experience more severe adverse events. Improper treatment of susceptible TB and poor access to quality drugs contribute to the development and transmission of RR/MDR-TB^3^.

Drug-resistant TB remains a significant public health concern, as many countries are experiencing low success rates in treating the disease. This highlights the urgent need for innovative treatment strategies that utilize fewer toxic drugs, that are plausible to administer, and demonstrate higher efficacy with increased treatment success rates^2,4^. According to data from global TB reports declared by the World Health Organization (WHO), only 56% of patients with RR and MDR-TB are effectively treated^5^. Based on the 2021 WHO-TB country profile, the overall TB incidence in Egypt was 10 cases per 100,000 population^6^. Additionally, the percentage of MDR-TB among new TB cases in Egypt was reported to be 2.2%^7,8^. In Egypt, the programmatic management of RR-TB began in 2006, with treatment success rates ranging from 57% in 2013 to 81% in 2010, and a median success rate of 64%^4,8–10^.

According to the 2013 WHO recommendations for MDR-TB treatment, bedaquiline (BDQ) is recommended for use in the treatment of RR/MDR-TB. The United States Food and Drug Administration (FDA) approved the use of BDQ to treat adult pulmonary MDR-TB combination therapy in December 2012^9^. BDQ is an innovative compound that belongs to the diarylquinoline chemical class. This new anti-TB medication features a quinolinic central heterocyclic core, with alcohol and amine side chains that are responsible for its antimycobacterial activity^10^. In particular, it stands out as the only anti-TB agent that acts on mycobacterial energy metabolism, effectively inhibiting mycobacterial adenosine triphosphate (ATP) synthase^11,12^. It is important to emphasize that the activity of BDQ is not restricted solely to drug-resistant MTB isolates; it also demonstrates efficacy against drug-susceptible strains. Moreover, it exhibits an effective half-life exceeding 24 h^12^.

Recently, the regimen that incorporates BDQ has been recommended as a priority treatment over the conventional regimen for patients with RR/MDR-TB^13,14^. This study hypothesized that a BDQ-containing regimen would achieve higher treatment success rates than conventional regimens for RR/MDR-TB patients in Egypt. It aimed to compare treatment success rates and their predictors, as well as the incidence of mortality and side effects, between patients receiving BDQ-containing regimens and those receiving conventional treatments.

## Subject and methods

### Study design and population

A multicenter prospective cohort study was conducted, including all patients diagnosed with TB who demonstrated resistance to first-line TB therapy, as confirmed by microbial sensitivity testing and GeneXpert. The study encompassed patients admitted to the Abbasya, Maamora, and Mansoura chest centers between April 1, 2020, and June 30, 2021. These three centers are the sole treatment facilities for RR/MDR-TB patients in Egypt, representing the entire patient population during this period. Follow-up continued until December 31, 2022. Eligible candidates for the BDQ regimen included those with contraindications, adverse events, or intolerance to any components of the conventional second-line regimen, as well as patients with confirmed resistance to fluoroquinolones or injectable drugs. Pregnant women and children under 18 years of age were excluded from the study^9,13^. All patients were continuously monitored for their electrolytes and electrocardiograms (ECGs).

### Study procedure

TB was diagnosed using Ziehl–Neelsen staining for sputum examination and culture on the Lowenstein-Jensen (LJ) medium. Initial diagnosis of RR/MDR-TB was conducted using rapid molecular diagnostic tests (e.g., GeneXpert), alongside conventional culture and drug susceptibility testing (DST) on LJ medium or automated liquid culture. For follow-up cases of MDR-TB, sputum smear microscopy and culture were performed at the start of treatment (baseline), monthly during the intensive phase (typically the first 6 months), and every 2–3 months during the continuation phase (typically the remaining 18 months). More frequent testing was warranted if there was clinical suspicion of treatment failure^15^.

Patients were provided with information regarding their treatment options and received counseling to aid them in making informed decisions. Social support was introduced to MDR-TB patients to assist them in adhering to their treatment regimen. Active drug safety monitoring (aDSM) was implemented for all patients. MDR-TB patients were categorized into two groups based on their eligibility for treatment. The BDQ group, comprising BDQ, clofazimine, linezolid, levofloxacin, and cycloserine. BDQ was administered at a recommended dose of 400 mg once a day orally for 14 days, followed by 200 mg three times a week for 22 weeks. The conventional second-line group, consisting of amikacin, levofloxacin, cycloserine, and ethionamide^16^. The end point to assess the study outcome was set after all enrolled patients completed their 18-month treatment course. At the end of treatment, the success rate for each group was calculated, including both cured patients and those who completed treatment^13^.

### Definitions of treatment outcomes

#### Cured

Treatment completed according to national policy without evidence of failure and three or more consecutive cultures are negative after the intensive phase, cultures taken at least 30 days apart^13,15^.

#### Completed

Treatment is completed according to national policy without evidence of failure and no record that negative cultures are taken^13,15^.

#### Defaulters (Loss to follow-up)

Individuals do not initiate treatment or stop their treatment regimen for a minimum duration of two months during the course of follow-up period^13,15^.

#### Death

Patients with TB who died for any reason before starting or during treatment^15^.

#### Treatment failure

A patient whose treatment regimen needs to be terminated or permanently changed to a new regimen or treatment strategy due to a positive sputum smear at month 5 during treatment. As stated by WHO guidelines, treatment failure includes several criteria. First, failure to culture conversion is indicated when sputum culture remains positive for MDR-TB bacilli after a defined period of treatment. Second, clinical deterioration is characterized by worsening respiratory symptoms or radiological findings despite adequate treatment adherence. Lastly, the acquisition of additional drug resistance refers to the development of resistance to additional anti-TB drugs during treatment^17^.

### Data management

Baseline data were collected for all RR/MDR-TB patients enrolled in treatment from 1st April 2020 to the end of June 2021. The information gathered included patients' gender, age, marital status, occupation, smoking history, and comorbidities such as human immune deficiency virus (HIV), hepatitis C virus (HCV), hepatitis B virus (HBV) infections, and diabetes mellitus. The data were entered into Excel sheets then validated and cleaned. All data were kept confidential and secure.

### Statistical analysis

Descriptive statistics were used to illustrate demographic characteristics, comorbidities, and treatment-related adverse events. This is achieved by employing measures such as the mean with standard deviation for normally distributed data, and the median with inter quartile range for skewed data. Primary outcome analysis examined difference between the BDQ-based treatment regimen and the conventional treatment regimen regarding the treatment success rate, by calculating the proportion of patients cured/completed in each group. The significant difference in the treatment success rate between the two groups was tested using the chi-square test and Fischer’s exact test.

To further investigate the factors influencing treatment success, a multivariable logistic analysis was conducted, adjusting for gender, age, presence of diabetes, smoking, weeks of treatment, and drug abuse. Kaplan–Meier survival curve provides estimates of survival probability over time (in weeks), categorized using a BDQ-inclusive regimen. The P-value was determined using the log-rank test, and the shaded region represents the 95% confidence interval.

Furthermore, the propensity score method was utilized to align the two groups based on age and sex, according to the study protocol. In total, 38 patients were successfully matched, and subsequent survival analysis was conducted. Additionally, another Kaplan–Meier survival curve assessed the probability of success over time (in days) within a matched group, categorized by the use of a BDQ-inclusive regimen. The Cox proportional hazards regression model was performed to determine the hazard ratio (HR) for treatment success within this matched group, accounting for variables such as lesion site, diabetes mellitus, HCV, and smoking.

### Ethics approval and consent to participate

The study was reviewed and approved by the Egyptian Ministry of Health and Population Research Ethics Committee [Com.No/ Dec. No: 19-2021/4]. The study was conducted in accordance with the guidelines outlined in the Declaration of Helsinki. All participants were required to sign an informed written consent form.

## Results

### Sociodemographic characteristics

Table 1 provides an overview of the baseline demographic and health-related characteristics. The study enrolled 84 participants. Among these individuals, the treatment protocol of 57 patients (67.8%) included BDQ. The median age of the studied participants was 39 years (IQR 32.0–52.0 years), and 22.6% were women. In terms of treatment initiation, more than half of the patients (52.4%) began their therapy in 2020, while the remaining 47.6% started in 2021. Half of the patients were smokers, 14.3% had diabetes mellitus, and 1.2% were co-infected with HIV and HCV. It is worth mentioning that 17.9% of the individuals had other comorbid conditions, with the majority (84.5%) diagnosed with RR-TB, and 42.9% categorized as new in the TB treatment regimen. Pulmonary TB was diagnosed in most of the patients (89.3%), while only 10.7% had extrapulmonary TB with lesion sites at cold abscess, peritoneal, and knee.

**Table 1:** Demographic, epidemiological, and health characteristics of 84 patients infected with tuberculosis (TB).

Variables	Bedaquiline included regimen			
	No (N = 27)	Yes (N = 57)	Total (N = 84)	P
Gender				0.601
Female	7 (25.9%)	12 (21.1%)	19 (22.6%)	
Male	20 (74.1%)	45 (78.9%)	65 (77.4%)	
Age (years)				0.803
Median	40	39	39	
IQR	32.0, 51.0	33.0, 52.0	31.0, 52.2	
Age category				0.059
18–44 years	17 (63.0%)	36 (63.2%)	53 (63.1%)	
45–64 years	8 (29.6%)	20 (35.1%)	28 (33.3%)	
≥ 65 years	2 (7.4%)	1 (1.7%)	3 (3.6%)	
Year of treatment initiation				0.310
2020	12 (44.4%)	32 (56.1%)	44 (52.4%)	
2021	15 (55.6%)	25 (43.9%)	40 (47.6%)	
Marital status				0.104
Married	13 (48.1%)	43 (75.4%)	56 (66.7%)	
Other*	14 (51.9%)	14 (24.6%)	28 (33.3%)	
Occupation				0.664
Employee/worker	8 (29.6%)	24 (42.1%)	32 (38.1%)	
Prisoner	1 (3.7%)	1 (1.8%)	2 (2.4%)	
Student	1 (3.7%)	1 (1.8%)	2 (2.4%)	
Unemployed/housewife	17 (63.0%)	31 (54.4%)	48 (57.1%)	
Number of previous treatment episodes				0.281
0	14 (51.9%)	22 (38.6%)	36 (42.9%)	
1	7 (25.9%)	25 (43.9%)	32 (38.1%)	
2	6 (22.2%)	10 (17.5%)	16 (19.0%)	
Resistance				0.163
RR-TB	24 (85.2%)	48 (84.2%)	72 (84.5%)	
MDR-TB	4 (14.8%)	9 (15.8%)	13 (15.5%)	
Number of household contacts				0.364
Mean ± SD	3.0 ± 2.1	3.4 ± 1.7	3.3 ± 1.8	
Previous contact with TB patient				0.691
No	25 (92.6%)	54 (94.7%)	79 (94.0%)	
Yes	2 (7.4%)	3 (5.3%)	5 (6.0%)	
Lesion site				0.002*
EXPTB	7 (25.9%)	2 (3.5%)	9 (10.7%)	
PTB	20 (74.1%)	55 (96.5%)	75 (89.3%)	
Patient category				0.152
New	14 (51.9%)	22 (38.6%)	36 (42.9%)	
Loss of follow-up Cat I/II	5 (18.5%)	4 (7.0%)	9 (10.7%)	
After the failure of Cat I/II	5 (18.5%)	22 (38.6%)	27 (32.1%)	
Relapse of Cat I/II	3 (11.1%)	9 (15.8%)	12 (14.3%)	
History of SLD, (No)	27 (100.0%)	54 (94.7%)	81 (96.4%)	0.221
HIV status, Negative	26 (96.3%)	57 (100.0%)	83 (98.8%)	0.140
Currently smoker	12 (44.4%)	30 (52.6%)	42 (50.0%)	0.482
Ex-smoker	1 (3.7%)	2 (3.5%)	3 (3.6%)	0.961
Alcoholism	1 (3.7%)	1 (1.8%)	2 (2.4%)	0.582
Drug abuse	5 (18.5%)	1 (1.8%)	6 (7.1%)	0.005*
Diabetes mellitus, (Yes)	4 (14.8%)	8 (14.0%)	12 (14.3%)	0.923
HCV, (No)	27 (100.0%)	56 (98.2%)	83 (98.8%)	0.481
HBV, (No)	27 (100.0%)	57 (100.0%)	84 (100.0%)	NA
Other Comorbidities	6 (22.2%)	9 (15.8%)	15 (17.9%)	0.471

Significant values are in [bold].

*Other includes widow, single, and divorced; EXPTB: Extrapulmonary TB, PTB: Pulmonary TB, IQR: Interquartile range, New: A patient who has either never received treatment for TB or has taken antituberculosis medication for less than four weeks, Cat I: Category I includes patients received a regimen of isoniazid, rifampicin, pyrazinamide, and ethambutol with or without streptomycin for 2 months, followed by isoniazid and rifampicin for 4 months, Cat II: Category II includes patients received isoniazid, rifampicin, pyrazinamide, ethambutol, and streptomycin for 2 months, followed by isoniazid, rifampicin, pyrazinamide, and ethambutol for 1 month, and then isoniazid, rifampicin, and ethambutol for 5 months, SLD: Specific learning disorders, HIV: Human immunodeficiency virus, HCV: Hepatitis C virus, HBV: Hepatitis B virus, NA: Not applicable, Variables presented as mean ± SD, median, and inter-quartile range, or number of patients number and percent the P value was calculated using a chi-squared or Fisher’s exact tests for categorical data or by Mann-Whitney or independent t-test for quantitative data, *P < 0.05 (significant).

### Adverse events and treatment outcomes

There were no reports of hearing impairments, renal failure, hypothyroidism, drug-induced hepatitis, convulsions, constipation, or cardiovascular side events among the registered participants. However, the incidence of skin discoloration was significantly higher in the BDQ-group compared to the conventional (38.6% versus 0.0%, P = 0.042). Regarding other adverse events, such as optic neuritis, allergy, gynecomastia, gastritis, nausea, vomiting, and diarrhea, there were no significant differences between the two groups (P > 0.05). A total of 55 patients (65.5%) completed the treatment regimen and were cured. Among the groups that included BDQ as a part of their treatment regimen, the success rate was (73.7%), while in the conventional group, the success rate was (48.1%), P = 0.042 Table 2.

**Table 2:** Treatment outcomes and the adverse events of 84 patients infected with tuberculosis.

Studied variables	Bedaquiline included regimen			P
	No (N = 27)	Yes (N = 57)	Total (N = 84)	
Gynecomastia	0 (0.0%)	1 (1.8%)	1 (1.2%)	0.491
Optic neuritis	0 (0.0%)	4 (7.0%)	4 (4.8%)	0.162
Skin rash	0 (0.0%)	3 (5.3%)	3 (3.6%)	0.233
Skin discoloration	0 (0.0%)	22 (38.6%)	22 (26.2%)	< 0.001*
Allergy	3 (11.1%)	4 (7.0%)	7 (8.3%)	0.532
Hyperuricemia	2 (7.4%)	1 (1.8%)	3 (3.6%)	0.191
Arthritis	5 (18.5%)	8 (14.0%)	13 (15.5%)	0.590
Psychosis	0 (0.0%)	1 (1.8%)	1 (1.2%)	0.492
Depression	0 (0.0%)	1 (1.8%)	1 (1.2%)	0.493
Peripheral neuritis	1 (3.7%)	9 (15.8%)	10 (11.9%)	0.111
Electrolyte disturbances	0 (0.0%)	1 (1.8%)	1 (1.2%)	0.494
Sleep disturbance	0 (0.0%)	1 (1.8%)	1 (1.2%)	0.490
Headache	0 (0.0%)	2 (3.5%)	2 (2.4%)	0.332
Diarrhea	1 (3.7%)	1 (1.8%)	2 (2.4%)	0.581
Vomiting	1 (3.7%)	1 (1.8%)	2 (2.4%)	0.582
Nausea	1 (3.7%)	1 (1.8%)	2 (2.4%)	0.581
Gastritis	0 (0.0%)	4 (7.0%)	4 (4.8%)	0.162
Treatment outcomes				0.042*
Treatment success	13 (48.1%)	42 (73.7%)	55 (65.5%)	
Treatment failure	8 (29.7%)	7 (12.3%)	15 (17.8%)	
Death (Yes)	6 (22.2%)	8 (14.0%)	14 (16.7%)	0.371

Significant values are in [bold].

Multivariate analysis revealed that except for the number of weeks of treatment (OR = 1.14 [95% CI, 1.06–1.27], P = 0.007) and BDQ-included regimen (OR = 3.97 [95% CI, 1.06–16.10], P = 0.042), all other variables were not significantly associated with an increased likelihood of treatment success Table 3.

**Table 3:** Multivariate logistic regression results for factors affecting treatment success rate.

Characteristic	OR^a^	95% CI^a^	P
Gender (male)	1.97	[0.38–11.20]	0.401
Age	1.04	[0.99–1.10]	0.112
Smoking	0.54	[0.09–2.67]	0.501
Drug abuse	0.84	[0.06–11.80]	0.902
Having diabetes mellitus	0.60	[0.10–4.31]	0.603
Weeks of treatment	1.14	[1.06–1.27]	0.007*
Bedaquiline included regimen	3.97	[1.06–16.10]	0.042*

^a^OR = Odds Ratio, CI = Confidence interval, the P was calculated using a chi-squared, *P < 0.05 (significant).

Significant values are in [bold].

**Figure 1:**
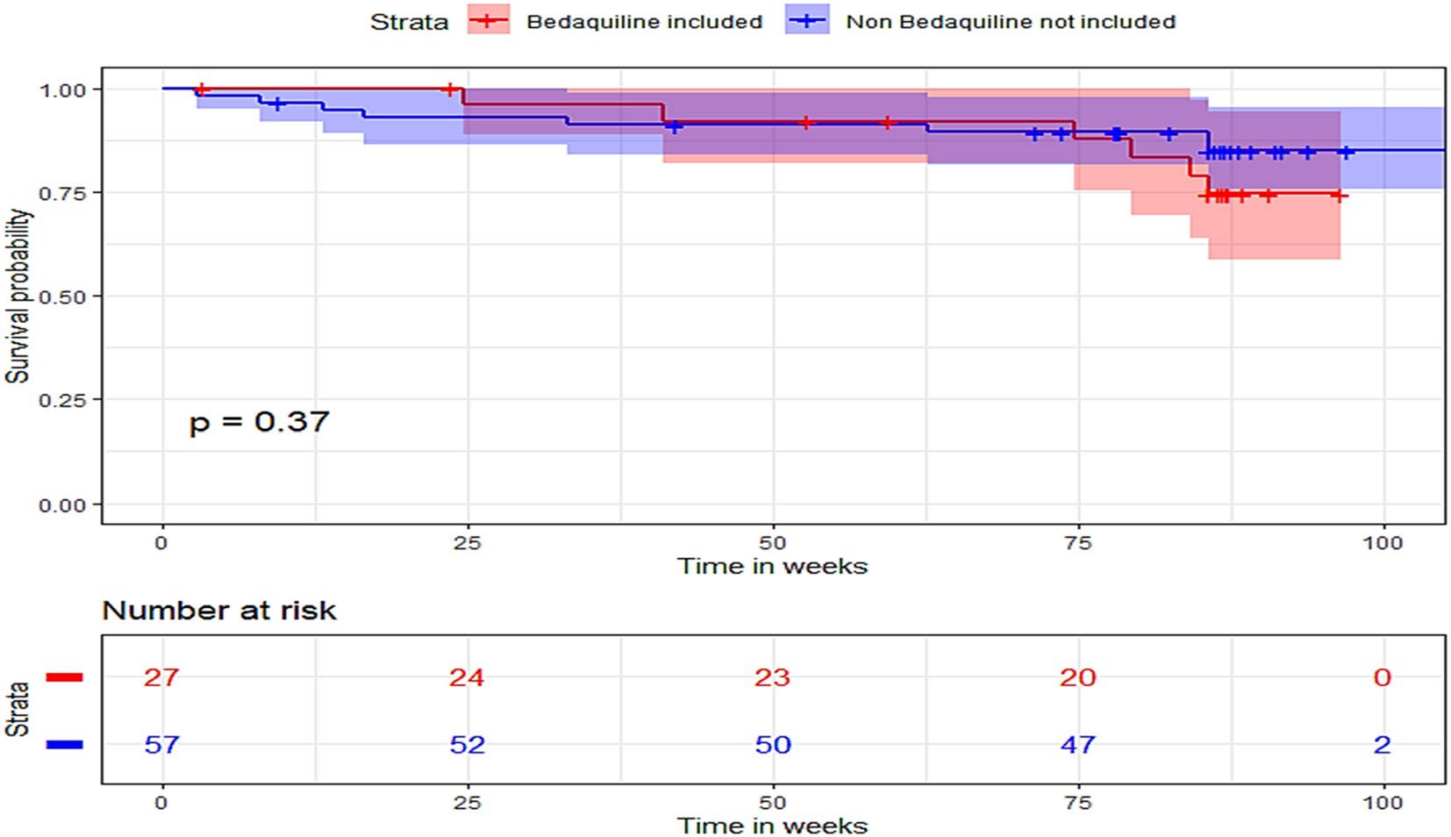
Kaplan–Meier survival curve estimates for the probability of survival as a function of time (in weeks), stratified by the administration of bedaquiline-inclusive regimen, The P was calculated with the log-rank test. The shaded area indicates 95% CI.

A total of 14 deaths occurred within 24 months of TB treatment initiation. Among the patients in the BDQ-included regimen group, the mortality rate was 14.0% (8 patients) versus 22.2% (6 patients) in the conventional group. The Kaplan–Meier survival curve demonstrated that overall mortality during the follow-up period was similar between the two groups, with a hazard ratio (HR = 0.62 [95% CI, 0.21–1.78], P = 0.372) Fig. 1.

### Propensity score, Success rate probability

**Figure 2:**
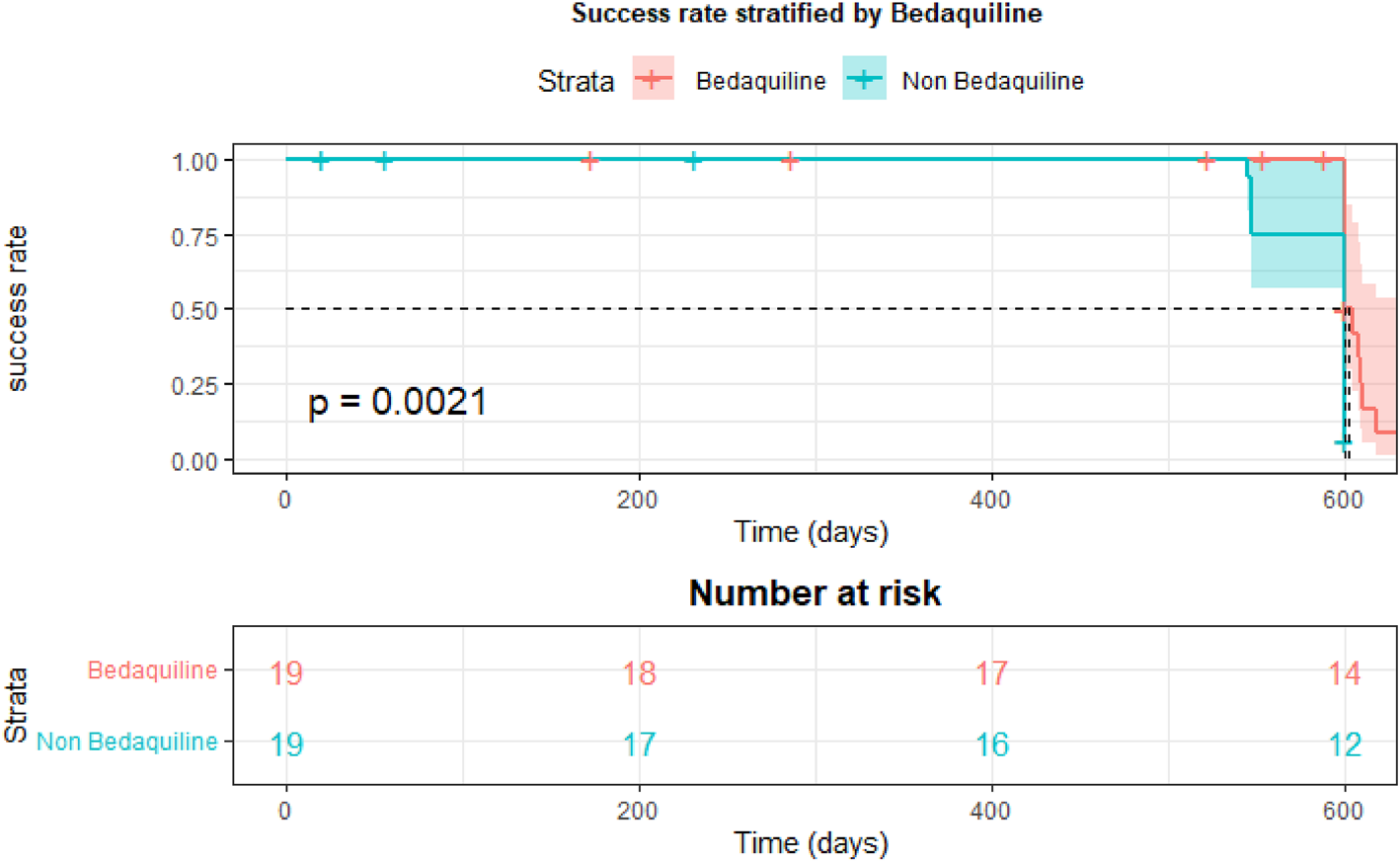
Kaplan–Meier survival curve estimates the probability of success rate as a function of time (in days) in the matched group, stratified by the administration of bedaquiline-inclusive regimen.

After matching by age and sex, an equal number of participants (n = 19) were included in the 2 groups. Kaplan–Meier curve revealed a noteworthy discrepancy in the median time to success between the two groups, with a P = 0.002. Upon performing Cox hazard regression and accounting for factors such as lesion site, diabetes mellitus, HCV, and smoking, it was observed that the addition of BDQ resulted in a statistically significant increase in the success rate, (HR = 6.79, [95% CI, 1.81–25.82] P = 0.005) Fig. 2 and Table S1.

## Discussion

The findings of this study indicate that of the 84 patients with RR/MDR-TB, two-thirds received the BDQ treatment regimen. There were statistically significant differences in treatment outcomes among the studied groups. While no major side effects were detected, significant incident cases of skin discoloration were documented in the BDQ group. The Kaplan–Meier survival curve revealed no statistical difference in mortality between the two treatment groups. Additionally, logistic regression identified weeks of treatment and the inclusion of BDQ in the regimen as significant factors affecting treatment success. Moreover, after propensity score matching by sex and age, the Kaplan–Meier survival analysis demonstrated a significant difference in the median time to success. Finally, a significant difference in the success rate between the two groups was documented by Cox hazard regression.

**Sex**: In this study, it is notable that men comprised the majority, accounting for 77.2% of patients with RR/MDR-TB. This finding is consistent with data from the WHO and other published studies, which have reported male percentages ranging from 62.0 to 76.0%^18–20^. These observations suggest that men may have a higher susceptibility to infection, possibly due to occupational exposure in densely populated areas and increased stress levels. Additionally, social, and economic factors might deter women from seeking timely medical consultations.

**Age**: the IQR of age within this study group was 32–52 years, reflecting the active period of occupation and physical stress. This age range closely aligns with the data reported in previous studies on MDR-TB, which falls between 35 and 43.5 years old^20–22^. It is noteworthy that a significant portion of patients in both groups were either unemployed or housewives, indicative of the lower socioeconomic status prevalent among these individuals. However, it is essential to emphasize that gender, age, marital status, and occupation did not have statistically significant effects concerning the treatment success.

**Comorbidities**: The risk of MDR-TB was increased in HIV-infected patients. HIV-infected patients experience gastrointestinal malabsorption, which affects the absorption of antimycobacterial drugs and leads to drug resistance. Additionally, multiple combination therapies prescribed for HIV-TB coinfected patients resulted in a reduction in treatment adherence and increased the possibility of drug resistance^23^. In this study, comorbidities such as diabetes mellites, HIV, and HCV infection were not statistically significantly associated with treatment success.

**Adverse events**: The adverse events of treatment can significantly impact treatment outcomes and prolong the duration of hospital stays. In this study, treatment in both groups was generally well tolerated, with mild and/or moderate adverse events that were not statistically significant. However, a higher incidence of skin discoloration, observed in 37.8% of the BDQ-group after completing the treatment follow-up, contrasting with a lower percentage reported in a study involving MDR-TB patients receiving the BDQ regimen, which showed 9.7%^24^. Conversely, a higher incidence of skin discoloration, reported in 95.1% of participants in a study conducted in India using BDQ combination therapy^25^. Skin discoloration is most probably due to the use of clofazimine in BDQ-group, a lipophilic compound with a lengthy half-life (approximately 72 h). Prolonged treatment results in its buildup in fatty tissues and organs. The accumulation of this phenazine dye in tissues leads to the skin acquiring a brown/orange pigmentation, attributed to drug-induced ceroid lipofuscinosis^26–28^.

Although prolongation of the QT interval has been previously reported in the literature as an adverse event from BDQ^23,29,30^, no such cases were observed in this study. Continuous monitoring of electrolytes and ECGs was conducted to enable early detection of such cardiovascular adverse events.

**Success rate**: In this study, the combination of BDQ with clofazimine, linezolid, levofloxacin, and cycloserine achieved a remarkable success rate (73.7%) after the follow-up period. This success rate contrasts starkly with the control group, which achieved a 48.1% success rate. It is worth noting that this success rate is nearly equal to that was reported from a multicenter study on 247 MDR-TB patients with a 71.3% success rate, with 58% in Africa and 71.8% in Europe^21^. Moreover in a study conducted among 383 patients from 29 countries the success rate was 74.2%^31^. Another study conducted on 91 patients from South Africa reported a success rate of 76%^29^. On the other hand, a retrospective cohort study on 5981 MDR-TB patients in South Africa reported a lower treatment success rate in the BDQ group at 66.9%, compared to our study, while the conventional group showed a similar outcome at 49.4%^32^. Regarding the conventional treatment success rate, in Egypt, a previous study on conventional second-line therapy for MDR-TB reported a nearly equal success rate of 52% in 577 MDR-TB patients^20^. Furthermore, another pooled study from five cohorts, involving 537 patients with MDR-TB treated with BDQ, showed a treatment success rate of 65.8%^33^. These results underscore the potential of the BDQ-containing regimen in achieving improved treatment outcomes by curing or completing the treatment regimen, especially compared to conventional therapies.

**Death rate**: The overall death rate in this study was 16.7%. It is noteworthy that the Kaplan–Meier survival curve revealed no statistically significant differences in mortality between the two groups. This observation is consistent with previous research^21,23^. The curve reveals that the mortality pattern in both groups was similar, with a HR of 0.62 and P = 0.372. In particular, the mortality rate in the BDQ-group was 14%, which resembles the results of a multicenter study that reported an overall mortality rate of 13.4%, with a significantly higher rate of 27.4% in African regions^21^. In contrast, another global cohort study documented an overall mortality rate of 6.5%, with striking disparities between continents, revealing a mortality rate of 23.9% in Africa and a lower rate of 3.5% in Europe^31^. These findings offer important insights into the safety of BDQ treatment, indicating that including it in the treatment plan doesn’t lead to a higher risk of mortality.

Using multivariable logistic regression analysis, the duration of treatment, as reflected in the weeks of treatment variable, emerged as a statistically significant predictor of treatment success. This implies that completing treatment durations was associated with an increased likelihood of positive treatment outcomes, underscoring the importance of extended therapeutic interventions in improving patient adherence and prognosis.

After propensity score matched by age and sex, the Kaplan–Meier survival curve revealed a substantial and statistically significant difference in the median time to success between the two groups. To gain deeper insight and control for potential confounders, a Cox hazard regression analysis was carried out. Factors such as lesion site, diabetes mellitus, comorbidities related to HCV, and smoking were carefully considered. Additionally, other studies documented that the Cox hazards model carried out on the presence of lung cavities and HCV infection showed a statistically significant treatment outcome and was associated with slowing the culture conversion^23,30^.

**Implication of this study**: Considering this comprehensive analysis, it is evident that incorporating BDQ into the treatment regimen significantly improves the success rate with minimal side effects. There were no significant differences in mortality or adverse effects, except for minor skin discoloration. This finding suggests that BDQ is a beneficial addition to the treatment protocol, enhancing patient outcomes without introducing major safety concerns.

### Strengths and limitations

This study revealed several notable strengths. Firstly, it encompassed all MDR-TB treatment centers in Egypt, enabling the data to be generalized to the broader Egyptian population. Secondly, the inclusion of control groups receiving conventional second-line therapy facilitates comparison between different treatment success rates. Thirdly, the utilization of the propensity score method was pivotal in ensuring alignment between the two groups. However, it is important to acknowledge that this study, being a cohort study, does not assess causality.

## Conclusions

This study has revealed that BDQ-based treatment regimens are associated with a significantly higher treatment success rate compared to conventional therapies. The duration of the regimen, particularly when it includes BDQ, significantly improves the treatment success rate. Although there was no statistically significant difference in the death rates between the two groups, the data suggest a potential positive impact of BDQ on treatment outcomes. These findings emphasize the potential of BDQ to improve the management of MDR-TB in Egypt and underscore the need for further research and monitoring to confirm these promising results.

### Supplementary Information

Supplementary Table S1.

## Data Availability

The datasets used and/or analyzed during the current study are available from the corresponding author on reasonable request.
