# Supplementary material for: Impact of bedaquiline regimen on the treatment success rates of multidrug-resistant tuberculosis patients in Egypt
Source: Sci Rep. 2024 Jul 15;14:16247. doi: 10.1038/s41598-024-65063-8 (PMC11250779; doi:10.1038/s41598-024-65063-8)
Supplement: Supplementary file 1 — Supplementary Table S1. [file 41598_2024_65063_MOESM1_ESM.docx]

| **Characteristic** | **HR***^1^* | **95% CI***^1^* | **P** |
| --- | --- | --- | --- |
| Bedaquiline use,Yes | 6.79 | [1.78 – 25.78] | **0.005*** |
| Lesion site (pulmonary) | 0.15 | [0.03 – 0.73] | **0.019*** |
| Smoking, Yes | 1.42 | [0.59 – 3.44] | 0.431 |
| Diabetes mellitus, Yes | 1.86 | [0.64 – 5.46] | 0.255 |
| HCV, Yes | 0.60 | [0.84 – 93.95] | 0.603 |
| Wald test = 14.49, on 5 df, p = 0.01  Log rank test = 20.81, on 5 df, p < 0.001 | | | |
| *^1^* HR= Hazard Ratio, CI = Confidence interval, *****P < 0.05 (significant). | | | |

Table S1: Cox hazard regression analysis for treatment success, after propensity matching by Age and Gender
